# *Rickettsia felis* meningoencephalitis in a child: a case report and literature review

**DOI:** 10.3389/fped.2026.1763281

**Published:** 2026-04-10

**Authors:** Xuehua Li, Yan Jiang, Rui Dai, Yue Yang, Weikai Wang

**Affiliations:** 1 The First Clinical Medical College of Gansu University of Chinese Medicine, Lanzhou, Gansu, China; 2 Department of Pediatric Intensive Care Medicine, Liangzhou Hospital, Wuwei, Gansu, China; 3 Department of Pediatrics, Gansu Provincial People’s Hospital, Lanzhou, Gansu, China; 4 Pediatric Intensive Care Unit (PICU), Gansu Provincial Maternity and Child Health Hospital (Gansu Central Hospital), Lanzhou City, Gansu, China

**Keywords:** case report, children, meningoencephalitis, next-generation sequencing (NGS), *Rickettsia felis*

## Abstract

*Rickettsia felis* (*R. felis*) infection occasionally invades the central nervous system, causing encephalitis or meningoencephalitis. Although the disease typically presents as mild to moderate illness, delayed diagnosis and treatment may increase the risk of adverse prognosis in pediatric patients. This article reports a case of *R. felis* meningoencephalitis in a child diagnosed by metagenomic next-generation sequencing (mNGS) of cerebrospinal fluid. mNGS analysis detected high-confidence *R. felis*-specific sequences, and potential background microbial contamination was effectively excluded through a bioinformatics pipeline, thereby providing critical evidence for etiological confirmation. Due to insufficient clinical awareness, limited pathogen detection methods, and the self-limiting nature of the disease, *R. felis* infection is prone to missed diagnosis and misdiagnosis in febrile children. The clinical manifestations are nonspecific; even with central nervous system involvement, routine laboratory tests are unlikely to suggest the microbial etiology, contributing to the underrecognition and underreporting of pediatric *R. felis* meningoencephalitis. Therefore, enhancing diagnostic awareness and achieving early precise diagnosis and treatment may help shorten the disease course and improve patient outcomes.

## 1 Introduction

*R. felis* is an emerging zoonotic pathogen associated with *Ctenocephalides felis* (1). It is an obligate intracellular Gram-negative bacterium that can be detected by Giemsa staining (2). The pathogen was first identified by electron microscopy in 1990 (3), and the first human case of infection was reported in the United States in 1994 (4), followed by a gradual increase in reports worldwide. In China, relevant cases have been documented since 2014 (5); however, no confirmed cases have been reported in northwestern China, including Gansu Province. Furthermore, current understanding of central nervous system infection caused by *R. felis* in children remains limited, with few related reports in the literature. Due to insufficient clinical awareness and diagnostic challenges, the disease is at high risk of missed diagnosis or misdiagnosis. Herein, we report a pediatric case of *R. felis* meningoencephalitis, describing its clinical manifestations, laboratory findings, and treatment outcomes, aiming to improve clinical recognition, diagnosis, and management of this disease.

**Figure 1:**
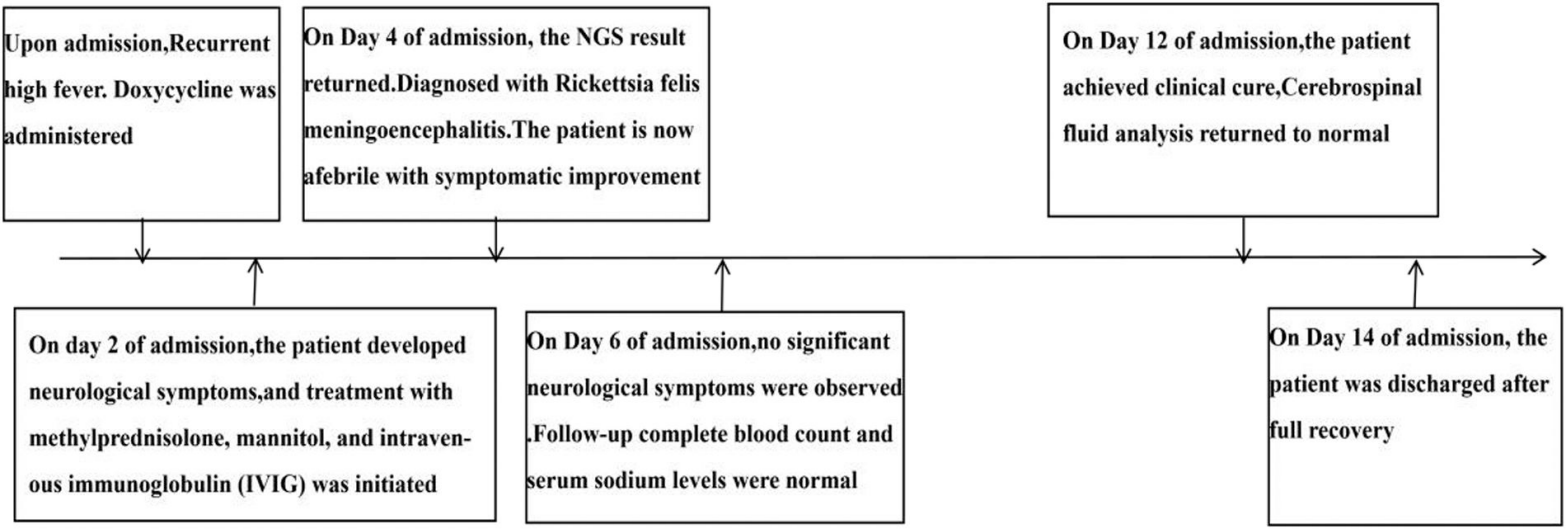
Clinical timeline of diagnosis, treatment, and recovery for a pediatric patient with *R. felis* Meningoencephalitis from admission to discharge.

## 2 Case presentation

A 9-year-old Han Chinese female, a resident of Pingchuan District, Baiyin City, Gansu Province, was admitted on September 10, 2025, with a 10-day history of intermittent fever and abdominal pain.

The patient developed fever without obvious trigger 10 days prior to admission, with a peak temperature of 40 °C, accompanied by lethargy, poor appetite, and dull periumbilical pain. No other significant symptoms were reported. An initial diagnosis of “acute upper respiratory tract infection” was made at a local hospital, and the patient received oral antibiotics and symptomatic treatment for 6 days without improvement. Subsequent laboratory investigations revealed leukopenia (2.56 × 10⁹/L), neutropenia (1.38 × 10⁹/L), mild thrombocytopenia (93 × 10⁹/L), C-reactive protein (CRP) <10 mg/L, and positive Mycoplasma pneumoniae antibody. Abdominal x-ray showed intestinal distension with superficial small air-fluid levels. Chest x-ray revealed bronchial inflammatory changes. A diagnosis of “Mycoplasma pneumoniae infection” was considered, and treatment with intravenous methylprednisolone sodium succinate for 1 day and azithromycin for 2 days was initiated. Due to lack of clinical improvement, the patient was transferred to our hospital.

The patient had been previously healthy, with no history of surgery, trauma, blood transfusion, or drug allergy. Routine immunizations were up to date. She was born at full term via spontaneous vaginal delivery with no perinatal complications and achieved all developmental milestones appropriately. Her parents are healthy and non-consanguineous, with no family history of hereditary disorders.

Epidemiological investigation revealed close contact with a stray cat 9 days prior to symptom onset. No definite flea bites were reported, and the cat had not undergone testing for parasitic or pathogenic infections. There was no recent travel history.

On admission, vital signs were as follows: temperature 36.9 °C, pulse 80 beats/min, respiratory rate 20 breaths/min, weight 26 kg, and oxygen saturation 98% on room air. The patient was alert but appeared unwell. Physical examination revealed multiple bilateral cervical lymph nodes, each approximately 2 cm in diameter, soft, non-tender, and mobile. No skin rashes were noted. Cardiopulmonary and abdominal examinations were unremarkable. Neurological examination revealed no meningeal signs, focal neurological deficits, or pathological reflexes.

Laboratory findings are summarized in Tables 1, 2.

**Table 1:** Results of blood routine, inflammatory markers, biochemistry, and etiological examinations for a pediatric patient with *R. felis* meningoencephalitis.

Parameter	Result	Reference	Range unit
Hematology & inflammation			
White blood cell count (WBC)	3.0	4.0–11.0	×10⁹/L
Neutrophil count (NEUT)	1.62	1.6–7.8	×10⁹/L
Platelet count (PLT)	161	100–300	×10⁹/L
reactive protein(CRP)	1.92	<10	mg/L
procalcitonin (PCT)	0.144	<0.065	ng/mL
Interleukin-6 (IL-6)	6.92	<7	pg/mL
Biochemistry			
Serum Sodium (Na^+^)	133.94	137–147	mmol/L
Hepatorenal function panel, myocardial enzyme panel	Within normal limits	-	-
Etiology			
Mycoplasma pneumoniae IgM	Positive	Negative	-
Epstein–Barr virus viral capsid antigen IgM/IgG(EBV-VCA IgM/IgG)	Positive	Negative	-
Epstein–Barr virus early antigen IgA(EBV-EA IgA)	Negative	Negative	-
Epstein–Barr virus DNA(EBV-DNA)	Negative	Negative	-
Respiratory pathogen nucleic acid panel, Brucella antibody,Mycobacterium tuberculosis-specific cellular immunity test, blood culture	All negative	Negative	-

**Table 2:** Results of conventional cerebrospinal fluid tests and next-generation sequencing (NGS) for a pediatric patient with *R. felis* meningoencephalitis.

Parameter	Result	Reference	Range unit
Opening pressure	210	80–200	mmH_2_O
Appearance	Colorless, clear	–	–
Total cells	38	–	/uL
Nucleated cells	36	–	/uL
Mononuclear cells	97	–	%
Total protein	0.52	0.1–0.4	g/L
Glucose	3.07	2.8–4.5	mmol/L
Cytology	Transformed lymphocytes: 95	–	%
Smear and culture	Negative	Negative	–
*R. felis*	7,636	–	Reads
Herpes simplex virus type 1 (HSV-1)	25	–	Reads

Metagenomic next-generation sequencing (mNGS) of cerebrospinal fluid (CSF) strictly adhered to laboratory quality control standards: total reads ≥ 300,000 (300 K); Q20 ≥ 90%; Q30 ≥ 85%; internal control (GAPDH) and pathogen capture efficiency (0.5) met the required thresholds; negative and positive controls were validated as acceptable.

Imaging Findings:echocardiography revealed slowed blood flow in both ventricles, presenting as the “spontaneous echo contrast” phenomenon. Given the child's *R. felis* infection and recurrent high fever, this sign was interpreted as a transient, functional alteration secondary to infection-related hemoconcentration and blood stasis. Chest computed tomography (CT) demonstrated mild thickening of the right interlobar fissure and localized emphysema in the medial basal segment of the right lower lobe. Abdominal and lymph node ultrasonography revealed a small amount of ascites (maximum depth: 13 mm) and bilaterally enlarged cervical and axillary lymph nodes with preserved normal architecture.

Non-contrast and contrast-enhanced CT and magnetic resonance imaging (MRI) of the brain revealed no significant abnormalities.

## 3 Diagnostic and treatment course

Upon admission, the patient presented with persistent high fever and chest CT findings of right lower lobe emphysema, with a positive Mycoplasma pneumoniae IgM antibody leading to an initial diagnosis of Mycoplasma pneumonia and the initiation of oral doxycycline. The following day, the patient developed neurological symptoms including dizziness, nausea, vomiting, and irritability without altered consciousness, and despite a repeated neurological examination showing no meningeal signs or focal deficits, an intracranial infection was suspected. A lumbar puncture was performed with cerebrospinal fluid (CSF) sent for metagenomic next-generation sequencing (mNGS), and empiric therapy was broadened to include intravenous methylprednisolone (1 mg/kg every 12 h for 2 days), mannitol, and intravenous immunoglobulin alongside symptomatic support. On day 4 of hospitalization, the patient became afebrile with significant neurological improvement, concurrent with CSF mNGS results detecting 7,363 sequence reads specific to *R. felis*. Integrating this finding with a history of potential *Ctenocephalides felis* exposure and a clinical course consistent with rickettsial disease, a final diagnosis of *R. felis* meningoencephalitis was established, and doxycycline was continued as targeted therapy. By day 6, laboratory parameters had normalized, and a follow-up CSF analysis on day 12 was unremarkable. The patient achieved full recovery and was discharged on day 14, with no neurological sequelae or evidence of recurrence observed during the 3-month follow-up period (see Figure 1).

## 4 Discussion

*R. felis* is an obligate intracellular Gram-negative bacillus (3) and an emerging flea-borne spotted fever group rickettsia (6). This pathogen has been detected in *Ctenocephalides felis* across more than 40 countries on five continents (7), with reported carriage rates ranging from 5% to 95% in different countries (8). *Ctenocephalides felis* serves as its primary vector (9). Human infection is presumed to occur mainly through the bite of infected *Ctenocephalides felis*, or via contact of broken skin with infected pet secretions or feces (10, 11).

Currently, the pathogenic mechanisms of *R. felis*, particularly regarding central nervous system (CNS) infection, remain poorly understood; current knowledge is largely extrapolated from studies on other Rickettsia species. It is hypothesized that the pathogen may bind to host cell receptors via surface proteins to invade and replicate intracellularly (12). It can invade vascular endothelial cells, triggering inflammatory responses, vascular injury (13, 14), and thrombosis (12), potentially leading to multi-organ dysfunction. Regarding CNS infection, it is speculated that the pathogen may invade the CNS by disrupting the integrity of the blood-brain barrier or increasing the permeability of the blood-cerebrospinal fluid barrier (15), leading to vascular endothelial cell damage, localized thrombotic vasculitis, and cerebral edema.

Clinical manifestations of *R. felis* infection are variable in severity and lack specificity (16), often mimicking other febrile illnesses (17). Literature reports indicate that common symptoms in children include fever, headache, and respiratory or gastrointestinal symptoms (18). The presenting symptoms in this pediatric case—fever, anorexia, and dull periumbilical pain—are consistent with these reports. Previous literature describes children with CNS involvement typically presenting with nausea, vomiting, lethargy, and seizures; photophobia, urinary incontinence, or meningeal signs may also occur (19, 20). Delayed diagnosis and treatment can lead to impairments in cognition, memory, and motor function (20). In this case, the child exhibited only dizziness, nausea, vomiting, and irritability, without seizures, altered consciousness, or positive neurological signs, indicating relatively mild CNS involvement.

Literature suggests that routine blood counts and serum sodium levels are often unremarkable in infected children. However, this patient developed leukopenia, neutropenia, mild thrombocytopenia, and hyponatremia during the disease course, findings consistent with reports in adult cases (21, 22). Cerebrospinal fluid (CSF) analysis typically reveals elevated opening pressure and protein, lymphocytic-predominant pleocytosis, with normal glucose and chloride levels (19, 20); the CSF findings in this child align with these reports. Furthermore, literature reports that brain MRI may show abnormal signals in the basal ganglia, frontoparietal lobes, and cerebral cortex, sometimes accompanied by meningeal thickening and enhancement; cranial CT may show hypodense areas in the cerebral cortex (19, 20). In this case, no imaging abnormalities were detected; the specific reasons for this warrant further investigation with accumulated cases.

Serological antibody testing is prone to cross-reactivity and typically yields positive results only after the first week of illness (23), limiting its utility for early diagnosis. Next-generation sequencing (NGS) of CSF offers high sensitivity and is of significant value for the early diagnosis of rare pathogens like *R. felis* (24). This case, like most previously reported cases, was confirmed by CSF NGS, highlighting the utility of this technique for early and precise diagnosis and timely treatment of CNS infections caused by *R. felis*.

Despite ongoing advances in etiological techniques, the diagnosis of central nervous system (CNS) infection caused by *R. felis* in children remains challenging. The primary reasons are as follows: (i) limited clinical awareness of the disease and its non-specific manifestations, which resemble those of viral encephalitis and autoimmune encephalitis (25), leading to a high risk of missed diagnosis or misdiagnosis; (ii) the absence of characteristic findings in routine laboratory tests, cerebrospinal fluid (CSF) analysis, or neuroimagings (17, 26), which hinders early definitive diagnosis based on conventional examinations; (iii)the limited availability of next-generation sequencing (NGS) in primary healthcare settings, further delaying early identification and confirmation.

Based on the patient's clinical presentations (fever and CNS involvement), etiological results, and the diagnostic criteria of the 21st edition of Nelson Textbook of Pediatrics (27), the following differential diagnoses were considered:

(1) Mycoplasma pneumoniae meningoencephalitis: Although M. pneumoniae IgM was positive, the absence of respiratory symptoms (e.g., cough), negative respiratory nucleic acid testing, and failure to detect the pathogen by CSF NGS, along with no response to azithromycin treatment, argued against acute infection. The positive IgM was considered a false positive or indicative of past infection.
(2) Epstein–Barr virus (EBV)-associated meningoencephalitis: EBV VCA-IgM/IgG were positive, but the patient lacked typical manifestations of EBV infection (e.g., hepatosplenomegaly, periorbital edema, rash), had normal liver function, and tested negative for EBV nucleic acid. CSF NGS also did not detect the virus. These findings did not support active infection; the serological results were considered possibly false positive or due to polyclonal activation.
(3) Herpes simplex virus (HSV) meningoencephalitis: The patient had a relatively mild clinical course with late-onset neurological symptoms and normal cranial magnetic resonance imaging. CSF NGS revealed only a low sequence count of HSV-1, and the neurological symptoms resolved rapidly without antiviral therapy. These features were inconsistent with typical HSV encephalitis, and the HSV detection was considered an incidental finding unrelated to the current illness.
(4) Autoimmune encephalitis: Autoimmune encephalitis typically requires high-dose pulse corticosteroid therapy followed by prolonged maintenance and gradual tapering. In this case, the patient's clinical symptoms improved rapidly following only short-term, low-dose corticosteroids and intravenous immunoglobulin (IVIG). Combined with the definitive etiological evidence, autoimmune encephalitis was considered unlikely.

Differential diagnoses for other febrile illnesses are detailed in the Supplementary Materials.

Regarding treatment, doxycycline penetrates the blood–brain barrier and is the first-line agent for CNS infections (28, 29). Short-term use (≤21 days) in children under 8 years of age has been proven safe and effective (28, 30, 31). While the optimal duration of therapy has not been standardized (ranging from a single dose to 15 days) (21), most patients show clinical improvement within two days of treatment (24). Given the moderate CNS penetration of doxycycline, administering a loading dose every 12 h during the initial 72 h has been recommended for severe cases to achieve rapid therapeutic effect (32); however, its superiority over conventional regimens requires further clinical validation. Minocycline, with better blood–brain barrier penetration, may serve as an alternative (33). In the present case, early empirical doxycycline was initiated due to suspected Mycoplasma infection, combined with corticosteroids and IVIG to alleviate CNS inflammation, and mannitol for intracranial pressure control. The patient's condition improved rapidly with a favorable outcome, which was attributed to early doxycycline administration and comprehensive supportive interventions.

This study has several limitations. First, etiological testing was not performed on the involved cat; thus, the route of infection and transmission chain were inferred solely from epidemiological clues and should be interpreted cautiously. Second, blood and respiratory specimens were not retained, precluding further validation of *R. felis* infection by polymerase chain reaction or NGS, which compromises the completeness of the diagnostic evidence. Third, EBV nuclear antigen (EBNA) testing was not conducted, making it difficult to definitively exclude false-positive serology or polyclonal reactivation and to clarify its potential role in disease progression. Fourth, only sporadic reports have suggested the possible efficacy of corticosteroids (34), and no standardized regimens or expert consensus exist regarding the use of corticosteroids and IVIG for *R. felis* CNS infection. These agents were administered as empirical adjunctive therapy in this case, and their definitive efficacy warrants further clinical investigation.

## 5 Conclusion

In recent years, alongside the rising prevalence of pet ownership and advancements in pathogen detection technologies, reported cases of *R. felis* infection have gradually increased; however, cases involving central nervous system (CNS) involvement in children remain relatively uncommon. The disease has an insidious onset, and its clinical manifestations and laboratory findings are often nonspecific, rendering it prone to missed diagnosis, misdiagnosis, and delayed treatment. In the present case, early recognition was hindered by insufficient awareness of the disease, and the diagnosis was ultimately established through cerebrospinal fluid next-generation sequencing (NGS) combined with epidemiological clues—underscoring the critical importance of a detailed exposure history and early implementation of pathogen detection. Therefore, in patients presenting with unexplained fever accompanied by cytopenia, suspected exposure to animals or ectoparasites, or unexplained CNS manifestations, *R. felis* infection should be considered. Early pathogen testing is essential to facilitate timely and accurate diagnosis and treatment, improve clinical outcomes, and reduce inappropriate antibiotic use and the emergence of antimicrobial resistance. Enhancing clinician awareness of this disease and strengthening public health education are of great clinical significance for the effective management of this emerging zoonosis.

## Data Availability

The datasets presented in this study can be found in online repositories. The names of the repository/repositories and accession number(s) can be found in the article/Supplementary Material.
