# Supplementary material for: Rickettsia felis meningoencephalitis in a child: a case report and literature review
Source: Front Pediatr. 2026 Apr 10;14:1763281. doi: 10.3389/fped.2026.1763281 (PMC13106386; doi:10.3389/fped.2026.1763281)
Supplement: Supplementary file 1 [file Datasheet1.pdf]

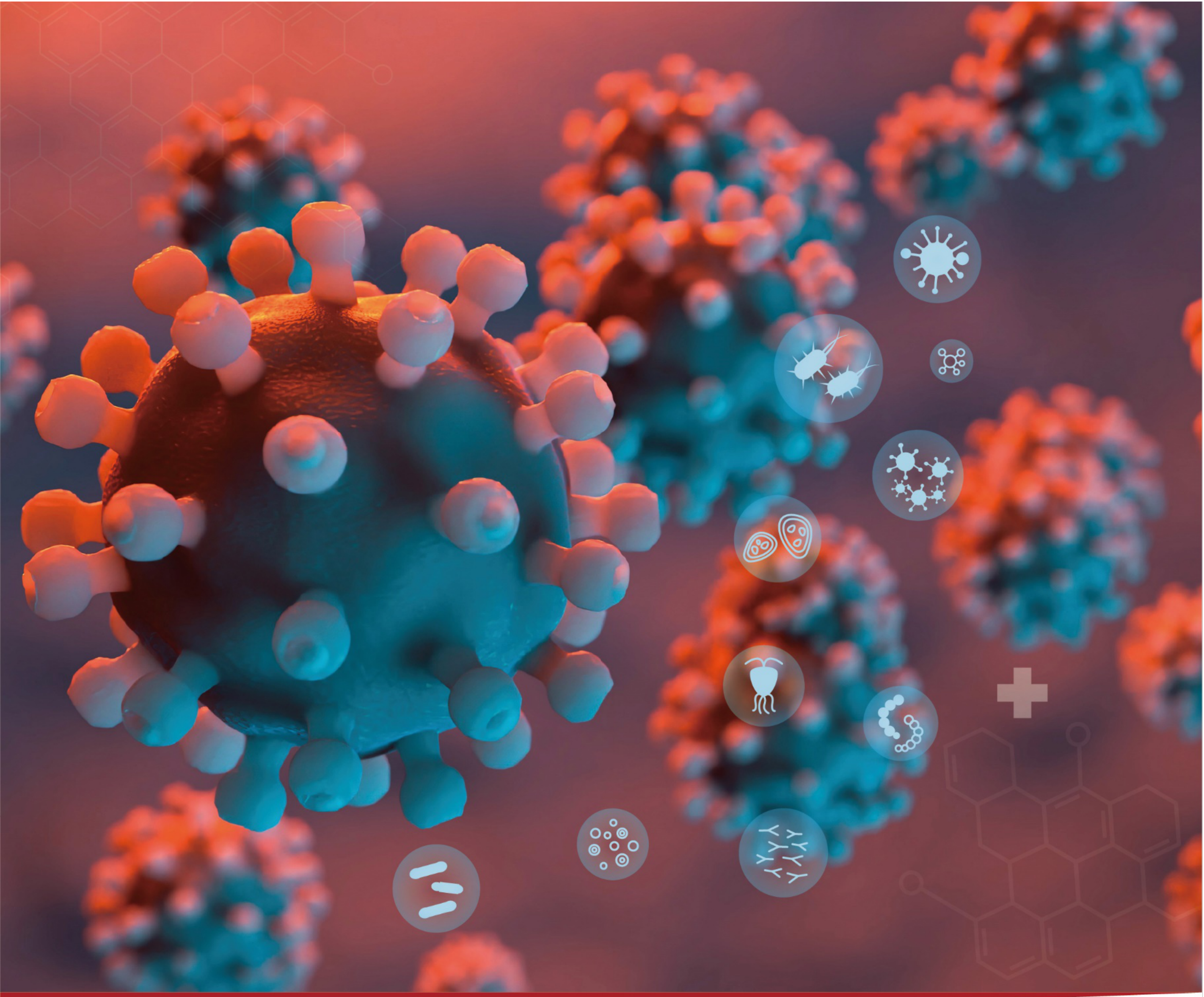

MetaCAP™病原微生物核酸高通量测序报告单

-----西安金域医学检验所

姓名:	李蔚	性别:	女	年龄:	9岁
医院:			科室:		
样本类型:	脑脊液		标本条码:	2719958721	
报告日期:	2025-09-15				

更多报告服务

金域医学
KingMed Diagnostics

MetaCAP™病原微生物核酸高通量测序报告单

受检者信息

姓名	李蔚	性别	女	年龄	9岁
送检医院				送检科室	
送检医生		住院号		床号	
标本类型	脑脊液	标本条码	2719958721	实验号	MC207
采样日期	2025-09-12	接收日期	2025-09-14	报告日期	2025-09-15
检测项目	MetaCAP病原微生物核酸高通量测序		检测方法	探针捕获高通量测序法	

临床信息

临床诊断					
主诉					
体温		白细胞计数		中性粒细胞数目/比例	
淋巴细胞数目/比例		单核细胞数目/比例		嗜酸粒细胞数目/比例	
红细胞沉降率		血小板计数		血红蛋白	
降钙素原		C反应蛋白		PCR	
G试验		GM试验		隐球菌荚膜多糖抗原	
镜检/培养			病原相关血清学检测		
影像					
前期抗感染方案及疗程					

MetaCAP病原微生物核酸高通量测序结果

细菌	真菌	病毒	寄生虫	耐药基因信息	毒力基因
猫蚤立克次体	未发现	单纯疱疹病毒1型(HSV-1)	未发现	未发现	未发现

* 对于免疫低下/缺陷患者，请注意关注微生态列表，建议综合考虑临床与其他检验结果进行确诊。

姓名:李蔚
条码:2719958721
实验号:MC207
样本类型:脑脊液

1.特殊病原体列表（分枝杆菌、支原体、衣原体、立克次体、螺旋体等）

属				复合群/种		
类型	名称	序列数	相对丰度	名称	序列数	置信度
G-	立克次体属 *Rickettsia*	7705	43.02%	猫蚤立克次体 *Rickettsia felis*	7636	99%

2.细菌列表

属				复合群/种		
类型	名称	序列数	相对丰度	名称	序列数	置信度
未检出						

3.真菌列表

属				复合群/种		
类型	名称	序列数	相对丰度	名称	序列数	置信度
未检出						

4.DNA/RNA 病毒列表

属				种/型/亚型		
类型	名称	序列数	相对丰度	名称	序列数	置信度
dsDNA	单纯疱疹病毒属 *Simplexvirus*	25	30.40%	单纯疱疹病毒1型(HSV-1) *Herpes simplex virus 1*	25	99%

5.寄生虫列表

属				种		
类型	名称	序列数	相对丰度	名称	序列数	置信度
未检出						

6.疑似人体微生态菌群列表

存在于人体皮肤、呼吸道、口腔、胃肠道、泌尿道的微生物，多为条件致病菌，正常条件下与人体共生，在免疫力低下/缺陷的患者中具有潜在致病性，请结合患者临床表现及其它辅助检查，综合判断。

属			复合群/种	
类型	名称	序列数	名称	序列数
未检出				

7.耐药基因列表

基因/位点	序列数	突变频率	疑似关联物种	药物
未检出				

8.毒力基因检出列表

毒力类别	毒力因子	毒力基因	序列数	疑似关联物种
未检出				

注释:

1.本项目参考CARD数据库，筛选临床关注的86种耐药基因家族进行检测，涵盖2907个耐药基因分型，包括CTXM、KPC、mecA等。
2.本项目可检测结核分枝杆菌、CMV、HIV、甲型流感病毒、乙型流感病毒、乙型肝炎病毒常见耐药基因核苷酸位点突变引发的耐药。
3.本项目参考VFDB数据库，筛选临床关注的64种毒力基因进行检测，包括高毒力肺炎克雷伯菌的rmpA、rmpA2、iutA，鲍曼不动杆菌pga A/B/C/D等。
4.研究表明，耐药基因、突变及毒力因子基因型与实际表型可能不完全一致，因此报告中的耐药基因、突变和毒力因子检出结果仅供参考，不能作为临床诊断或用药的唯一依据，请综合患者情况谨慎使用检测结果。

病原体解释说明(附病原基因组覆盖图)

猫蚤立克次体

Rickettsia felis

该菌是革兰阴性菌，隶属于立克次体属，属于过渡群，是胞内寄生细菌，分布于地球上除南极以外的各大洲，可存在于蜱虫、跳蚤、虱子和其他节肢动物，是人畜共患病病原体，可引起跳蚤传播斑疹热（FBSF）。

Rickettsia felis 基因组覆盖图(覆盖率:17.76%)

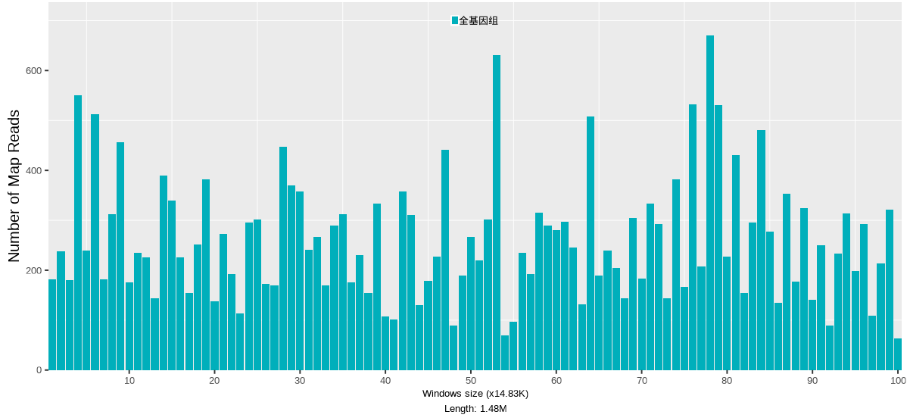

单纯疱疹病毒1型(HSV−1)

Herpes simplex virus 1

该病毒是DNA病毒，隶属于α疱疹病毒亚科，单纯疱疹病毒属，又称为人类疱疹病毒1型（HHV−1），发生在世界各地，没有季节性，HSV1感染比HSV2感染的频率更高且更早，通过与分泌物中的病毒直接接触传播，主要引起口腔−面部感染，也可以引起生殖器感染、脑炎、角膜炎感染等。

Herpes simplex virus 1 基因组覆盖图(覆盖率:1.26%)

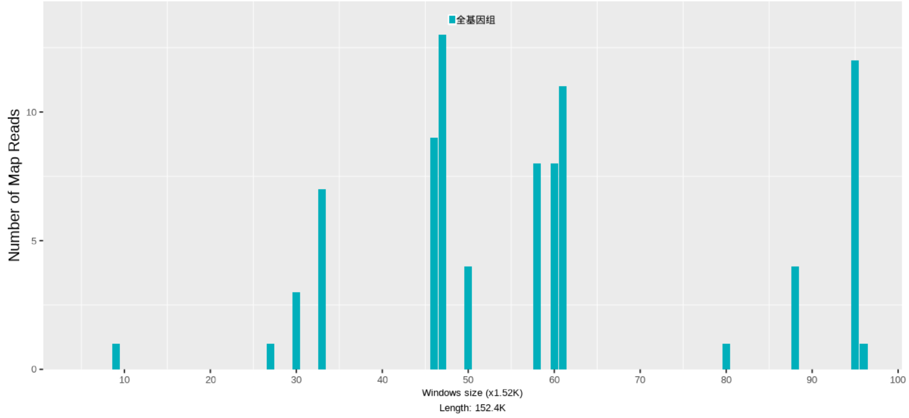

名词解释

1. **序 列 数**： 指将总序列数标准化至一百万条reads后，能够高质量比对到该病原体的序列数目(RPM)。
2. **相对丰度**： 将病原体按照细菌、真菌、病毒和寄生虫进行分类，相对丰度是该病原体相应分类中序列数的相对比例。
3. **置 信 度**： 结合物种检出序列数、排名、特异性验证等指标综合判断的得分，分值范围为0-100，分值越高代表该病原体检测结果越可信。
4. **耐药基因**： 指可能会导致病原体产生耐药性的基因序列。
5. **毒力基因**： 指可能会导致细菌产生毒力的基因序列。

参考文献

1.Michael M J , Binnicker M J , Sheldon C , et al. A Guide to Utilization of the Microbiology Laboratory for Diagnosis of Infectious Diseases: 2018 Update by the Infectious Diseases Society of America and the American Society for Microbiology [J]. Clinical Infectious Diseases, 2018, 67(6):813-816.
2.Chiu C Y , Miller S A . Clinical metagenomics [J]. Nature Reviews Genetics, 2019, 20(6): 341-355.
3.Byrd A L , Belkaid Y , Segre J A . The human skin microbiome [J]. Nature Reviews Microbiology, 2018, 16(3).
4.He T , Kaplan S , Kamboj M , et al. Laboratory Diagnosis of Central Nervous System Infection [J]. Current Infectious Disease Reports, 2016, 18(11):35.
5.Miller S , Naccache S N , Samayoa E , et al. Laboratory Validation of a Clinical Metagenomic Sequencing Assay for Pathogen Detection in Cerebrospinal Fluid [J]. Genome Research, 2019, 29(5): 831-842.
6.Huffnagle G B , Dickson R P , Lukacs N W . The Respiratory Tract Microbiome and Lung Inflammation: A Two-way Street [J]. Mucosal Immunology, 2017, 10(2):299-306.
7.Mamanova, L., Coffey, A., Scott, C. et al. Target-enrichment strategies for next-generation sequencing. Nat Methods 7, 111–118 (2010).

质控信息

质控参数	数据量	捕获效率	Q30比率	内参
质控结果	合格	合格	93.20%	合格

检测技术说明

病原捕获宏基因组检测技术是一种不依赖于临床培养的可检测不同样本类型中微生物组的高通量测序方法，该技术基于宏基因组学，通过百万级别的定制化特异性探针与样本中的微生物核酸进行杂交，对目标区域的序列进行捕获、富集以及高通量测序。

MetaCAPTM可检测21388种微生物，包括11958种细菌(其中包括180种分枝杆菌、251种支原体/衣原体/立克次体/螺旋体)、7373种病毒(其中包括4414种RNA病毒，2959种DNA病毒)、1714种真菌和343种寄生虫。重点捕获1292种细菌、517种真菌、1380种病毒、210种寄生虫、86个耐药基因的2627个耐药分型以及74个毒力基因。适用于各种样本类型的病原微生物种属信息及耐药毒力基因检测。具有超广谱、高灵敏、兼容性强的优势，助力感染精准诊疗。

结果中所列的病原微生物以原核微生物、病毒、真菌、寄生虫进行分类，检测结果仅对该样本的本次检测负责。检测结果中提供的检出序列数可在一定程度上体现样本中相应微生物的相对丰度，不等同于样本中该微生物的绝对含量。

本检测非常规临床检测项目，目前主要用于辅助临床诊断目的，不作为最终诊断依据，以上检测结果需由临床医师结合其他临床证据进行综合判断。此外，同其他检测方法一样，基因检测亦存在由于技术、样本以及操作所致低概率的假阴性或假阳性的风险。

声明：本检测仅对来样负责，如果对结果有疑义，请在报告发布后3天内与我们联系，多谢合作！

更多报告服务	主检	郭帅	审核	余玉荣	XA003MXZ0BVZL1S
	主检实验室：西安金域<br>临床咨询电话：15332383997		网址：HIDE&		收样点:HIDE&
	地址：西安市经开区尚稷路8989号C座11层<br>本报告系委托西安金域检测<br>如需了解更多请关注微信服务号“金域服务”				传真：020-22283222

常见病原微生物检测列表

细菌

金黄色葡萄球菌	血链球菌	肺炎克雷伯菌	艰难梭菌	布鲁氏菌	鲍曼不动杆菌
表皮葡萄球菌	牛链球菌	催产克雷伯菌	脆弱拟杆菌	鼠疫耶尔森菌	卡他莫拉菌
溶血葡萄球菌	屎肠球菌	奇异变形杆菌	具核梭杆菌	小肠结肠炎耶尔森菌	嗜水气单胞菌
人葡萄球菌	粪肠球菌	普通变形杆菌	小韦荣球菌	炭疽芽孢杆菌	豚鼠气单胞菌
路邓葡萄球菌	脑膜炎奈瑟菌	阴沟肠杆菌	痤疮丙酸杆菌	蜡样芽孢杆菌	产单核细胞李斯特菌
沃氏葡萄球菌	淋病奈瑟菌	黏质沙雷菌	齿双歧杆菌	贝纳柯克斯体	伊氏放线菌
腐生葡萄球菌	大肠埃希菌	摩根摩根菌	迟钝真杆菌	汉氏巴尔通体	牛型放线菌
头葡萄球菌	痢疾志贺菌	霍乱弧菌	产黑色素普雷沃菌	五日热巴通体	内氏放线菌
藤黄微球菌	福氏志贺菌	副溶血性弧菌	结核分枝杆菌	多杀巴斯德菌	黏液放线菌
肺炎链球菌	鲍氏志贺菌	拟态弧菌	麻风分枝杆菌	白喉棒状杆菌	龋齿放线菌
化脓链球菌	宋内志贺菌	河流弧菌	堪萨斯分枝杆菌	百日咳鲍特菌	星形诺卡菌
无乳链球菌	伤寒沙门菌	创伤弧菌	鸟-胞内分枝杆菌	嗜肺军团菌	巴西诺卡菌
缓症链球菌	副伤寒沙门菌	幽门螺杆菌	脓肿分枝杆菌	铜绿假单胞菌	鼻疽诺卡菌
草绿色链球菌	肖氏沙门菌	破伤风梭菌	龟分枝杆菌	空肠弯曲菌	
口腔链球菌	希氏沙门菌	产气荚膜梭菌	偶发分枝杆菌	胎儿弯曲菌	
唾液链球菌	肠炎沙门菌	肉毒梭菌	流感嗜血杆菌	嗜麦芽窄食单胞菌	

病毒

甲型流感病毒	鼻病毒	轮状病毒	发热伴血小板减少综合征病毒	人疱疹病毒7型	埃博拉病毒
乙型流感病毒	腺病毒	诺如病毒	克里米亚-刚果出血热病毒	人疱疹病毒8型	人类免疫缺陷病毒
副流感病毒	2019新型冠状病毒	星状病毒	单纯疱疹病毒1型（HSV-1）	流行性乙型脑炎病毒	人类嗜T细胞病毒
呼吸道合胞病毒	SARS冠状病毒	甲型肝炎病毒	单纯疱疹病毒2型（HSV-2）	登革病毒	狂犬病病毒
麻疹病毒	普通冠状病毒	乙型肝炎病毒	水痘－带状疱疹病毒(VZV)	森林脑炎病毒	人乳头瘤病毒
人腮腺炎病毒	脊髓灰质炎病毒	丙型肝炎病毒	EB病毒 (EBV)	西尼罗病毒	B19病毒
人偏肺病毒	柯萨奇病毒	丁型肝炎病毒	巨细胞病毒 (CMV)	汉坦病毒	博卡病毒
风疹病毒	肠道病毒	戊型肝炎病毒	人疱疹病毒6型	痘病毒	

真菌

絮状表皮癣菌	石膏样小孢子菌	卡氏枝孢霉	粗球孢子菌	热带念珠菌	烟曲霉
石膏样毛癣菌	秕糠马拉色菌	疣状瓶霉	皮炎芽生菌	光滑念珠菌	镰刀菌
红色毛癣菌	球形马拉色菌	甄氏外瓶霉	巴西副球孢子菌	近平滑念珠菌	毛霉
铁锈色小孢子菌	申克孢子丝菌	链格孢霉	马尔尼菲篮状菌	库德里阿兹威毕赤酵母(克柔念珠菌)	耶氏肺孢子菌
犬小孢子菌	裴氏丰萨卡菌	荚膜组织胞浆菌	白念珠菌	新型隐球菌	

寄生虫

恶性疟原虫	间日疟原虫	颚口线虫	棘阿米巴原虫	隐孢子虫	链状带绦虫
三日疟原虫	刚地弓形虫	贝尔蛔虫	溶组织内阿米巴	等孢子虫	
卵形疟原虫	管圆线虫	阿米巴原虫	环孢子虫	蓝氏贾第鞭毛虫	

支原体/衣原体/立克次体/螺旋体

肺炎支原体	解脲脲原体	恙虫病东方体	鹦鹉热衣原体	苍白密螺旋体(梅毒螺旋体)
人型支原体	普氏立克次体	沙眼衣原体	问号钩端螺旋体	伯氏疏螺旋体
生殖支原体	斑疹伤寒立克次体	肺炎衣原体	回归热疏螺旋体	奋森疏螺旋体

人体皮肤常见正常菌落

（Allyson L.Byrd,et al，Nature Reviews Microbiology，2018）

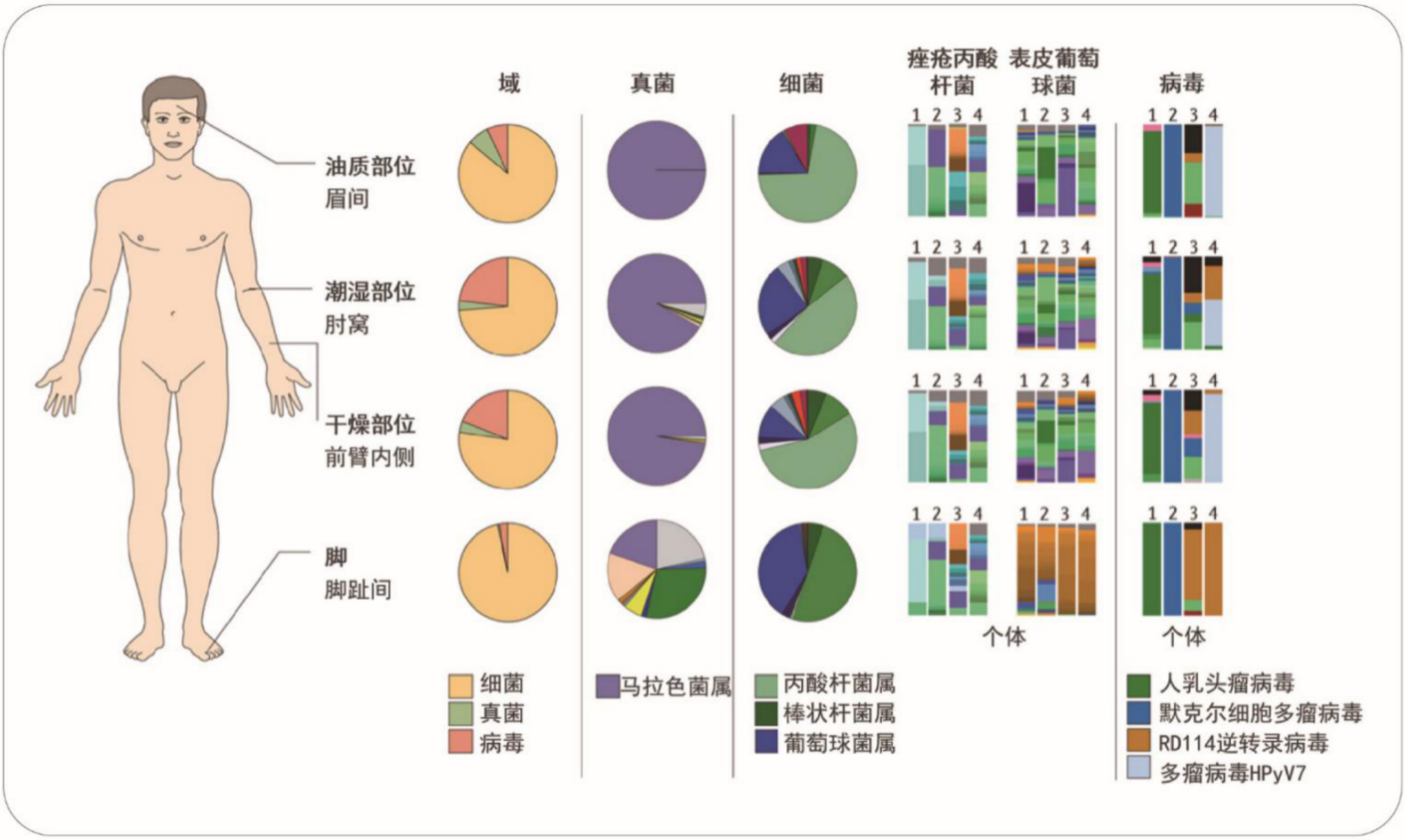

	手掌，前臂	鼻孔，肘窝，腹股沟折痕，腘窝	鼻翼，脸颊，眉间，外耳道，耳后，胸骨柄部，背部	脚趾间，脚趾甲，脚后跟
细菌	· 痤疮丙酸杆菌<br>· 结核硬脂酸棒状杆菌<br>· 缓症链球菌<br>· 口腔链球菌<br>· 假肺炎链球菌	· 结核硬脂酸棒状杆菌<br>· 人葡萄球菌<br>· 痤疮丙酸杆菌<br>· 表皮葡萄球菌<br>· 头葡萄球菌	· 痤疮丙酸杆菌<br>· 表皮葡萄球菌<br>· 结核硬脂酸棒状杆菌<br>· 头葡萄球菌	· 结核硬脂酸棒状杆菌<br>· 人葡萄球菌<br>· 沃氏葡萄球菌<br>· 表皮葡萄球菌<br>· 头葡萄球菌
真菌	· 限制性马拉色菌<br>· 球形马拉色菌<br>· 塔宾曲霉<br>· 近平滑念珠菌<br>· 合轴马拉色菌	· 球形马拉色菌<br>· 限制性马拉色菌<br>· 合轴马拉色菌<br>· 塔宾曲霉	· 限制性马拉色菌<br>· 球形马拉色菌<br>· 合轴马拉色菌	· 限制性马拉色菌<br>· 红毛癣菌<br>· 球形马拉色菌<br>· 须毛癣菌
病毒	· 传染病软疣病毒<br>· 丙酸杆菌噬菌体<br>· 默克尔细胞多瘤病毒<br>· 多瘤病毒HPyV7<br>· 人乳头瘤病毒（β）	· 传染病软疣病毒<br>· 丙酸杆菌噬菌体<br>· 多瘤病毒HPyV6<br>· 默克尔细胞多瘤病毒<br>· 多瘤病毒HPyV7	· 丙酸杆菌噬菌体<br>· 传染病软疣病毒<br>· 默克尔细胞多瘤病毒<br>· 多瘤病毒HPyV6<br>· 人乳头瘤病毒（γ）	· 丙酸杆菌噬菌体<br>· 默克尔细胞多瘤病毒<br>· 人乳头瘤病毒（μ）<br>· 人乳头瘤病毒（β）

人体中枢神经系统感染常见病原

（Taojun He，et al，Curr Infect Dis Rep，2016）

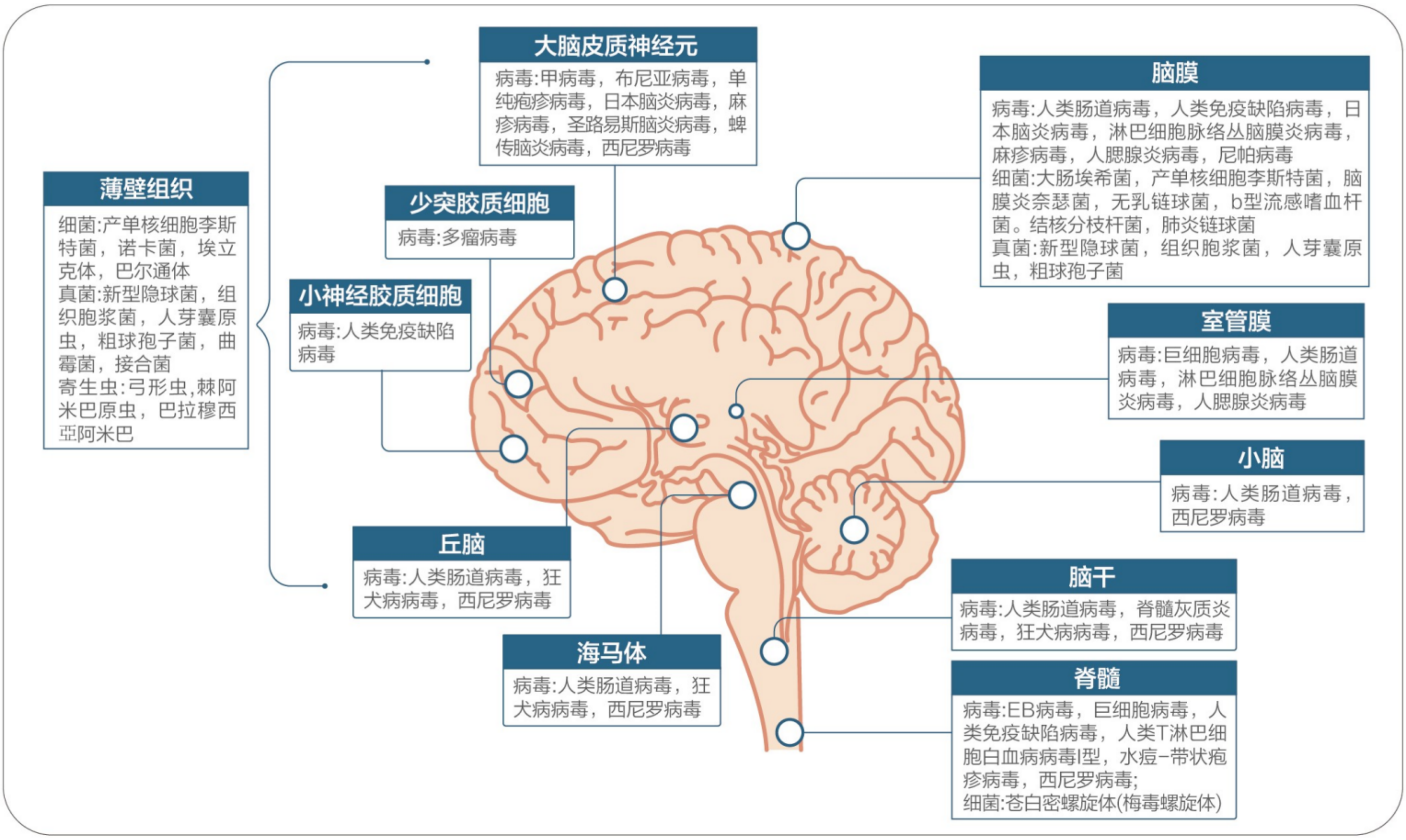

	原核微生物	真菌	病毒	寄生虫
脑炎	产单核细胞李斯特菌，诺卡菌属，埃希菌属，巴尔通体属，人型芽孢杆菌，衣原体	新型隐球菌<br>曲霉属<br>酵母菌	腺病毒，单纯疱疹病毒1型，单纯疱疹病毒2型，巨细胞病毒，人疱疹病毒-6，人疱疹病毒-7，细小病毒B19型，流感和副流感病毒，人腮腺炎病毒，尼帕和亨德拉病毒，狂犬病病毒，西尼罗病毒，虫媒病毒，肠道病毒	弓形虫<br>变形虫
脑膜炎	大肠埃希菌，产单核细胞李斯特菌，脑膜炎奈瑟菌，无乳链球菌，流感嗜血杆菌，结核分枝杆菌，肺炎链球菌，人型芽孢杆菌，衣原体	新型隐球菌	腺病毒，单纯疱疹病毒1型，单纯疱疹病毒2型，水痘－带状疱疹病毒，虫媒病毒，人腮腺炎病毒，尼帕和亨德拉病毒，淋巴细胞脉络丛脑膜炎病毒，肠道病毒	
脊髓炎	结核分枝杆菌<br>苍白密螺旋体(梅毒螺旋体)		水痘-带状疱疹病毒，巨细胞病毒，EB病毒，人类嗜T细胞病毒-1，人类嗜T细胞病毒-2，西尼罗病毒	

人体呼吸道常见正常菌落

（GB Huffnagle，et al，Mucosal Immunol，2018）

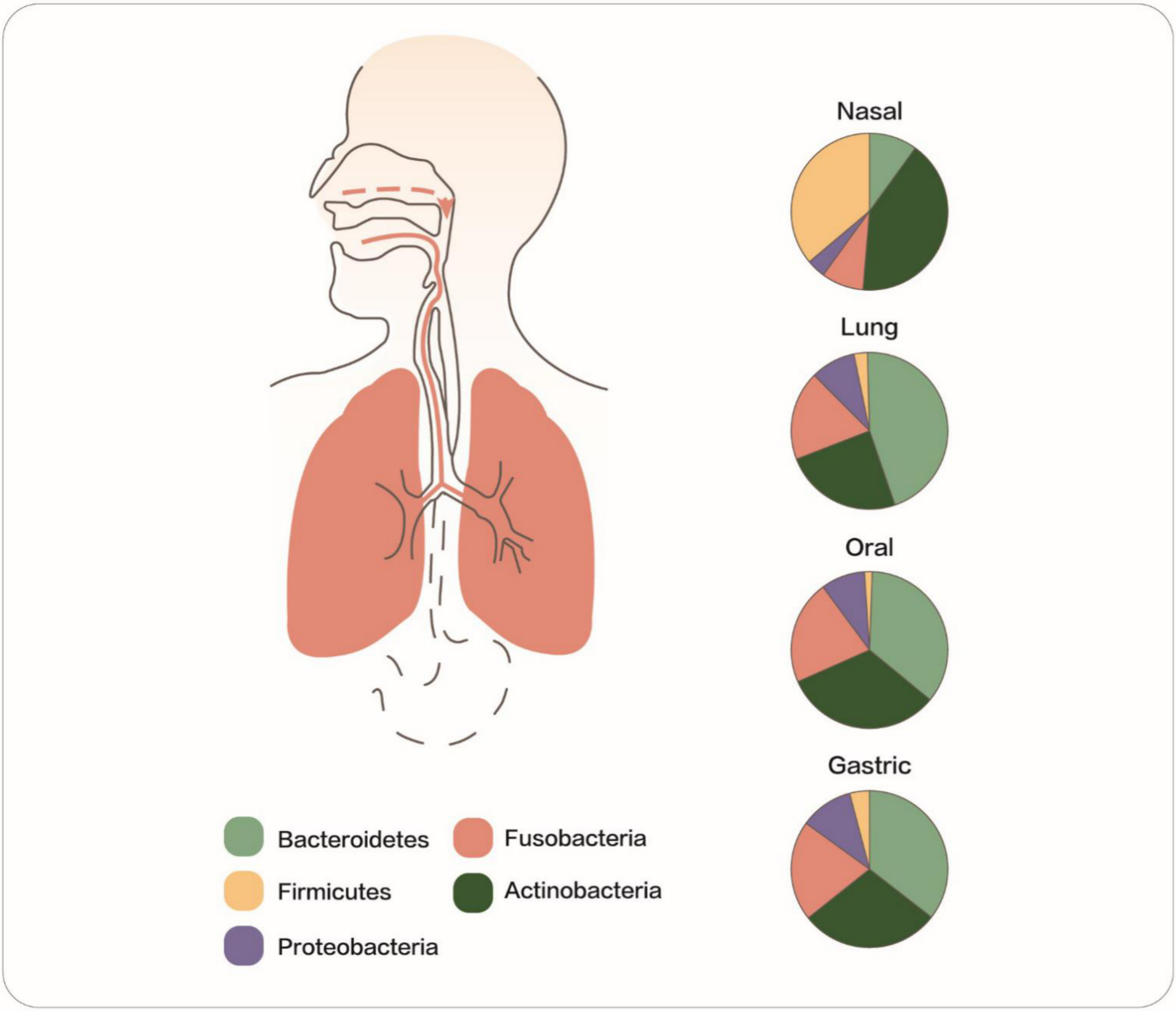

	葡萄球菌属	丙酸杆菌属	棒状杆菌属	莫拉菌属	链球菌属	嗜血杆菌属	罗氏菌属	奈瑟菌属	纤毛菌属	厌氧菌
鼻腔	√	√	√	√						
口腔			√		√	√	√	√	√	√
肺					√	√				

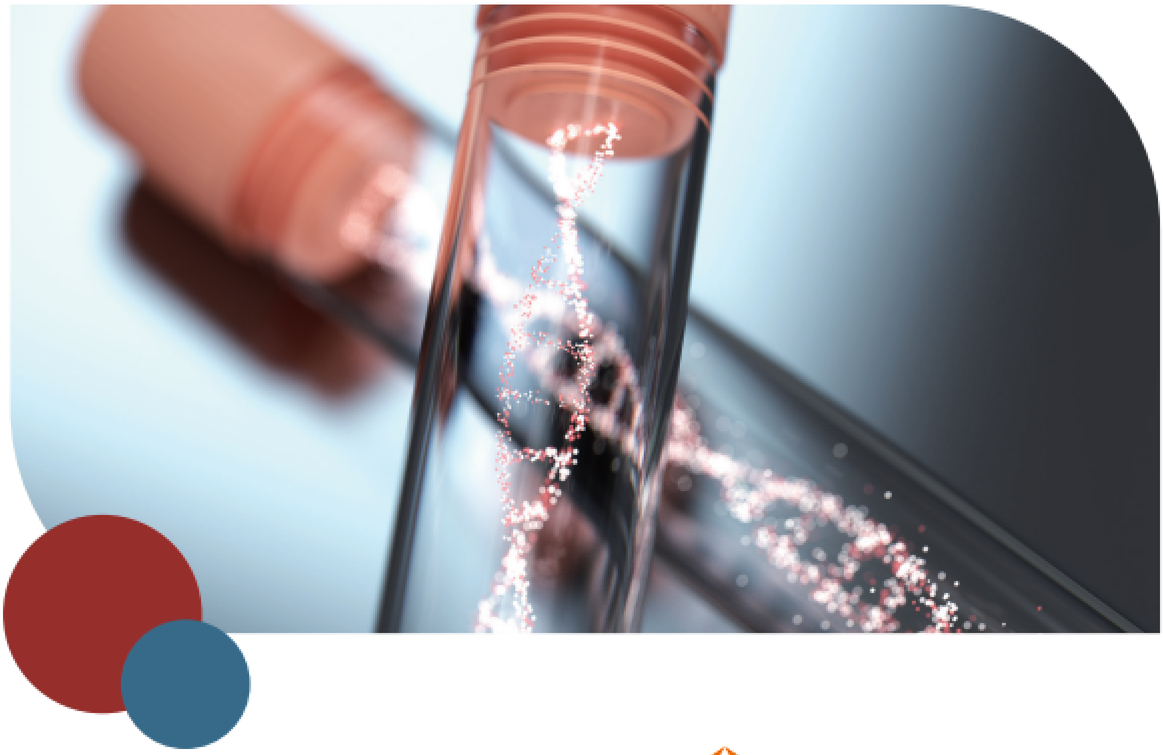

MetaCAP™ 病原微生物核酸高通量测序

———广谱、DR共检、超敏经济，实现病原微生物检测的新突破

广州金域医学检验集团股份有限公司 ☎4001-111-120 www.kingmed.com.cn

广州（总部）丨北京丨天津丨上海丨重庆丨香港丨澳门丨深圳丨呼和浩特丨乌鲁木齐丨银川丨南宁丨拉萨丨杭州丨合肥丨福州丨南昌丨济南丨青岛丨郑州丨武汉丨长沙丨海口丨博鳌丨长春丨石家庄丨太原丨沈阳丨哈尔滨丨西宁丨南京丨昆明丨西安丨成都丨贵阳丨毕节丨兰州
