# Supplementary material for: Rickettsia felis meningoencephalitis in a child: a case report and literature review
Source: Front Pediatr. 2026 Apr 10;14:1763281. doi: 10.3389/fped.2026.1763281 (PMC13106386; doi:10.3389/fped.2026.1763281)
Supplement: Supplementary file 2 [file Datasheet2.pdf]

Differential diagnoses for other febrile illnesses are detailed in the supplementary materials

Based on the patient's clinical manifestations (history of cat contact, fever, lymphadenopathy, cytopenia, and neurological symptoms) and the diagnostic criteria of the 21st edition of Nelson Textbook of Pediatrics, the following conditions were considered in the differential diagnosis and subsequently excluded:

(1) Infectious mononucleosis: Positive for EBV-CA-IgM/IgG; however, the absence of pharyngitis, hepatosplenomegaly, and eyelid edema, along with normal lymphocyte count, no atypical lymphocytes, and negative EBV-DNA, excluded this diagnosis.

(2) Cat scratch disease: Despite a history of cat contact, the absence of primary cutaneous lesions, non-regional lymphadenopathy, and failure to detect Bartonella henselae in cerebrospinal fluid (CSF) by next-generation sequencing (NGS) argued against this diagnosis.

(3) Anaplasmosis/Ehrlichiosis: With no history of tick bite or blood transfusion, and negative findings on blood smear and CSF NGS for relevant pathogens, these infections were excluded.

(4) Brucellosis: No history of contact with cattle or sheep, along with negative Rose Bengal test and blood culture, ruled out brucellosis.

(5) Pulmonary tuberculosis: Chest CT revealed no tuberculous lesions; the tuberculosis-specific cellular immune test was negative, and Mycobacterium tuberculosis was not detected in CSF by NGS, excluding active pulmonary tuberculosis.

(6) Systemic juvenile idiopathic arthritis (SJIA): The absence of arthritis and characteristic rash, together with normal inflammatory markers, provided no support for SJIA.

(7) Malignancy (e.g., lymphoma): No malignant features were noted on lymph node ultrasound, and the clinical course and treatment response were inconsistent with malignancy.
